# A new species and new records of *Engytatus* from the Hawaiian Islands (Heteroptera, Miridae)

**DOI:** 10.3897/zookeys.796.21054

**Published:** 2018-11-15

**Authors:** Dan A. Polhemus

**Affiliations:** 1 Department of Natural Sciences, Bishop Museum 1525 Bernice Street, Honolulu, HI 96817 USA Department of Natural Sciences Honolulu United States of America

**Keywords:** *
Engytatus
*, Hawaii, new species, new records

## Abstract

*Engytatushenryi***sp. n.** is described from the Waianae Mountains of Oahu. This new species feeds on *Abutilonsandwicense* (Malvaceae), an endangered understory plant in mesic forests. A dorsal habitus photograph and line drawings of key male genitalic structures are provided for *E.henryi*, accompanied by a photograph of the host plant. *Cyrtopeltiskahakai* Asquith is given a new generic assignment as *Engytatuskahakai* (Asquith) new combination, and additional locality and host-plant records are provided for four other Hawaiian endemic *Engytatus* species.

## Introduction

The genus *Engytatus* is represented by 28 species worldwide, and has undergone a modest insular radiation in the Hawaiian Islands, where 9 endemic species have been previously known to occur, most of them single-island endemics (Perkins 1912, Carvalho and Usinger 1960, Gagné 1968, Asquith 1992). These species occur on host-plant species in the genus *Cyrtandra* in the Gesneriaceae; *Dubautia* in the Asteraceae; *Lysmachia* in the Primulaceae; *Phyllostegia* in the Labiatae; *Scaevola* in the Goodeniaceae; and *Sida* in the Malvaceae. In the course of general Heteroptera surveys in the Waianae Mountains of Oahu, the author discovered yet another new species in this insular assemblage, present on *Abutilonsandwicense*, another host plant in the Malvaceae. This new species is described below, and additional geographic and host-plant records are provided for four other native Hawaiian *Engytatus* species. In particular, it is shown that individual *Engytatus* species utilize multiple host species in the genus *Cyrtandra* on Oahu, and multiple species of *Dubautia* on Maui, indicating that species isolating mechanisms operate primarily at the host-plant genus level in Hawaiian *Engytatus*.

## Methods

All measurements in the descriptions below are given in millimeters, and were made using a Wild M3Z dissecting microscope equipped with an ocular micrometer. High resolution dorsal habitus photographs were taken using an AutoMontage digital imaging system linked to a Leica M165-C dissecting stereomicroscope, with post-processing using Photoshop software. Line drawings of male genitalic structures were made using a camera lucida attached to a Wild M3Z dissecting microscope.

Synonymies provided under species are nomenclatural only, rather than comprehensive for all previous citations in the literature. For material collected by the author, CL numbers following localities refer to a collection locality-numbering scheme used to cross-reference photographs and other metadata to specific collecting localities.

Nomenclature for host plants follows Wagner et al. (1999). Host plant determinations were verified by consultation with botanists at the Bishop Museum, and checked against voucher specimens in the Herbarium Pacificum at that institution. In cases where the botanical names provided on original host-plant labels for the *Engytatus* specimens examined have now been superseded due to more refined taxonomic interpretations, the currently accepted host-plant name is provided in brackets following the name originally used on the label. Collection locality elevations originally taken with an altimeter reading in feet have also been converted to metric values in brackets.

The following abbreviations are used for specimen depositories:

**BPBM**Bernice P. Bishop Museum, Honolulu, Hawaii, USA.

**USNM**United States National Museum of Natural History, Smithsonian Institution, Washington, DC, USA.

## Taxonomy

### Genus Engytatus

Taxon classificationAnimaliaHemipteraMiridae

Reuter, 1876

#### Discussion.

The Hawaiian species currently held in *Engytatus* were all originally described in the genus *Cyrtopeltis* (Perkins 1912, Carvalho and Usinger 1960, Gagné 1968), within which *Engytatus* was considered a subgenus by most authors, although Zimmerman (1948) treated it as a full genus and placed the Hawaiian species described at that time within it. Cassis (1986), in his doctoral dissertation, subsequently elevated all subgenera of *Cyrtopeltis*, including *Engytatus*, to full genus status, a taxonomic arrangement subsequently followed in the catalog of Schuh (1995), thus validating Zimmerman‘s previous interpretation. Asquith (1992) described yet another Hawaiian *Cyrtopeltis* species, but gave no subgeneric assignment, and made no comment regarding his decision to use this genus name in preference to *Engytatus*. In the current work, all Hawaiian species formerly assigned to *Cyrtopeltis* are considered to fall within the generic limits of *Engytatus* as it is currently interpreted. In addition to the endemic Hawaiian species, another widespread *Engytatus* species, *E.modestus* (Distant), has also been introduced to the Hawaiian Islands, where it is a pest of tomato and other agricultural crops (Tanada and Holdaway 1954).

Following a modest amount of targeted collecting and taxonomic scrutiny from 1930–1968, Hawaiian *Engytatus* species have been infrequently collected or discussed in the scientific literature over the past 45 years. However, more recent records for previously described species, listed below, as well as the discovery of a new species, as reported herein, indicate that these insects are still present, even on the heavily developed island of Oahu, in areas of native forest. Overall, *Engytatus* species seem to be generally overlooked due to their inconspicuous habits and specialized associations with increasingly rare host plants.

### Engytatus henryi sp. n.

Taxon classificationAnimaliaHemipteraMiridae

http://zoobank.org/4046845E-2F99-4EE4-A86B-041E2FE1303C

#### Description.

**Figure 1.**
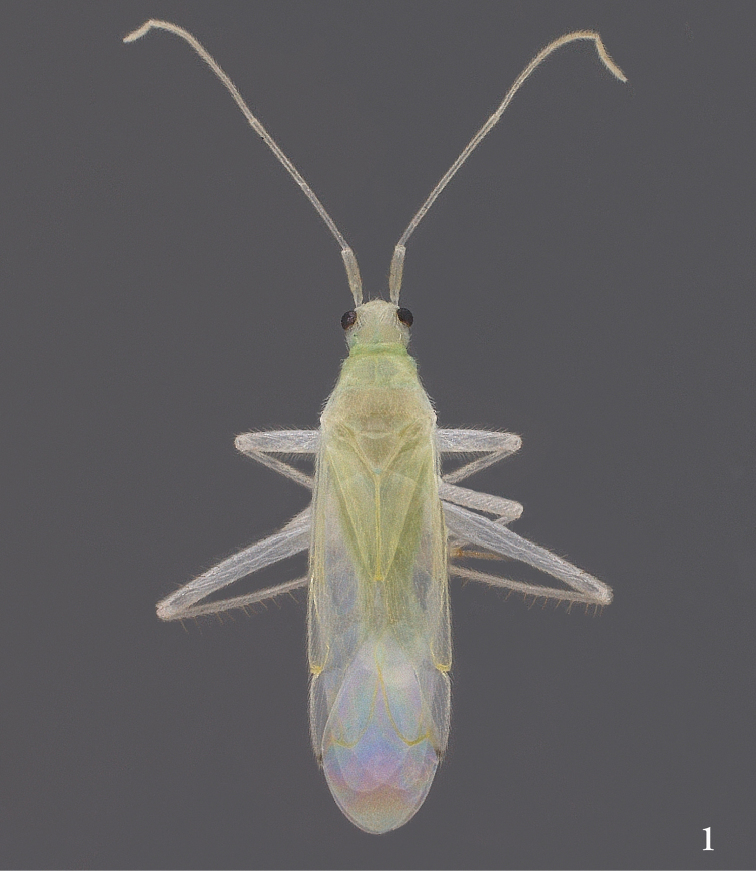
*Engytatushenryi* sp. n., male, dorsal habitus photograph. Specimen from Kaluaa Gulch, Waianae Mountains, Oahu.

*Male* with general form slender, elongate, parallel-sided (Fig. 1); overall length 3.90–4.10, length from tip of tylus to cuneal fracture 2.60–2.80, maximum width (across base of cuneus) 1.00–1.05. General coloration pale yellowish green, with base of head, anterior margin of pronotum, and entire abdomen bearing more saturated green to bluish-green coloration.

*Head* length 0.30, width across eyes 0.52, pale yellowish green with more saturated bluish-green coloration at posterolateral margins; eyes relatively small, dark reddish-black, length 0.15, width 0.10; vertex width 0.32, bearing scattered moderately long, semi-erect, pale setae. Antennae long, slender, all segments very pale yellowish, segment I bearing 3 erect, golden, spinose setae, all segments thickly covered with short, semi-erect pale setae, lengths of segments I–IV = 0.40, 1.10, 1.15, 0.50. Rostrum length 1.25, reaching apices of middle coxae, pale golden yellow, extreme tip dark.

*Pronotum* length 0.60, width 0.80, pale yellowish green, bearing scattered semi-erect pale setae. *Scutellum* length 0.40, width 0.35, pale yellowish-green, bearing scattered semi-erect pale setae.

*Hemelytra* translucent, uniformly pale yellowish green except extreme posterior apex of cuneus brown (Fig. 1); entire hemelytral surface set with simple, semi-recumbent pale setae; wing membrane very pale grey, veins yellowish green.

*Legs* slender, elongate (Fig. 1), very pale yellow, tarsi pale golden brown; all leg segments clothed with very short, pale, recumbent setae; anterior margins of all femora bearing ~10 evenly spaced, slender, erect, spine-like setae; posterior margin of fore femur with numerous slender, erect, pale setae; posterior margins of middle and hind femora each with 3–4 very long, slender, erect pale setae, lengths of setae subequal to greatest width of corresponding femur on which they occur; anterior margin of hind tibia with scattered long, erect, spine-like setae, lengths of setae ~2× the tibial width.

*Ventral surface* predominantly pale green, clothed with short, recumbent pale setae, these setae becoming longer and more numerous adjacent to genital cavity.

**Figures 2–7.**
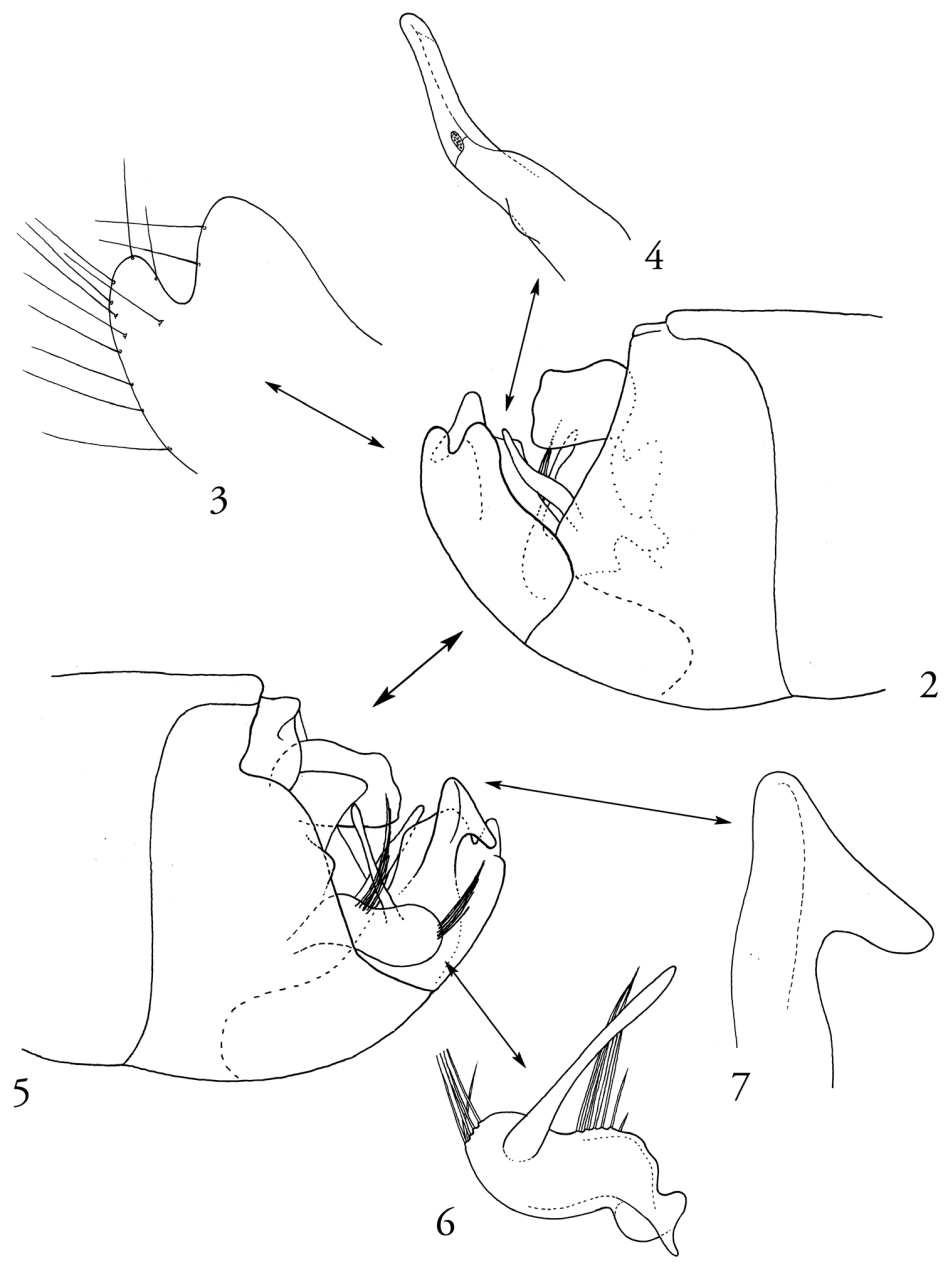
*Engytatushenryi* sp. n., male genitalic structures. Specimen from Kaluaa Gulch, Waianae Mountains, Oahu. **2** Male terminal abdomen, right lateral view **3** Terminal processes on right side of male pygophore, right lateral view **4** Male right paramere, right lateral (outer) view **5** Male terminal abdomen, left lateral view **6** Male left paramere, right lateral (inner) view **7** Terminal process on left side of male pygophore, left lateral view.

*Male genitalia* with right paramere slender and finger-like (Fig. 4); left paramere stout and bilobate basally, basal lobe bearing acuminate tuft of long, dark setae, distal lobe with slender, elongate, darkly sclerotized process, apex of distal lobe with acuminate tuft of long setae (Fig. 6); proctiger with two small apical lobes on right side when viewed laterally (Figs 2, 3), left side with larger, hook-like process (Figs 5, 7).

*Female* length overall length 4.20–4.30, length from tip of tylus to cuneal fracture 2.90–3.00, maximum width (across base of cuneus) 1.05–1.10; similar to male in general structure and coloration, but slightly more yellowish.

#### Host plant.

*Abutilonsandwicense* (O. Degener) Christophersen (Malvaceae).

#### Types.

Holotype, male, HAWAIIAN ISLANDS, Oahu, Waianae Mountains, middle section of Kaluaa Gulch, W. of Schofield Barracks, 1600 ft. [485 m.], 21°27'49"N, 158°06'34"W, 26 April 2017, CL 8527, D. A. Polhemus (BPBM). Paratypes: HAWAIIAN ISLANDS, Oahu: 10 males, 16 females, same data as holotype (BPBM, USNM).

#### Etymology.

The name “henryi” is a patronym honoring Dr. Thomas J. Henry for his many years of scientific effort devoted to the study of Heteroptera, particularly Miridae.

#### Discussion.

*Engytatushenryi* runs to *E.cyrtandrae* in the key of Gagné (1968), by virtue of its parallel-sided form, pale dorsal pubescence, elongation of the head behind the eyes, uniformly pale antennae, and pale-colored body and wings with only a small dark mark at the extreme apex of the cuneus (Fig. 1). It differs from *E.cyrtandrae* in its larger size, with the overall length across both sexes being 3.90–4.10 mm, versus 3.13–3.28 mm in *E.cyrtandrae*; by having a much different set of structures at the apex of the pygophore, consisting of two small, rounded lobes on the right side (Figs 2, 3) and a large, hooked lobe on the left (Figs 5, 7), rather than a pair of more developed processes on the right side, one acuminate and the other bulb-like, as in *E.cyrtandrae* (see figs 5a–b in Gagné 1968); and by the shapes of the male parameres (Figs 4, 6).

**Figure 8.**
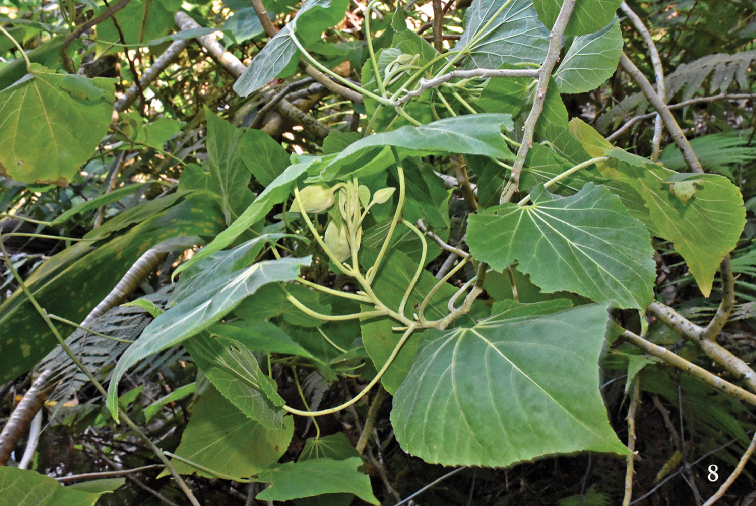
*Abutilonsandwicense*, host plant for *Engytatushenryi* sp. n.; photograph taken at type-locality in middle Kaluaa Gulch, Waianae Mountains, Oahu.

*Ecologicalnotes*. The type series of *E.henryi* was taken from a stand of *Abutilonsandwicense* (Fig. 8) in a fenced enclosure along the middle reach of Kaluaa Gulch, on the windward side of the Waianae Mountains in western Oahu. *Abutilonsandwicense* is a sprawling to arborescent, large-leaved shrub that was formerly common in the understory of Hawaiian mesic forests, but has been badly reduced in extent by wildland fire and the depredations of feral pigs, such that it is now listed as Endangered under the federal Endangered Species Act.

### Engytatus kahakai

Taxon classificationAnimaliaHemipteraMiridae

(Asquith) comb. n.

Cyrtopeltis kahakai Asquith, 1993: 17.

#### Discussion.

In his description of *C.kahakai*, Asquith (1993) did not provide a subgeneric placement within *Cyrtopeltis* or otherwise discuss his generic assignment. In the assessment of the author, *C.kahakai* is clearly congeneric with the remainder of the endemic Hawaiian dicyphine radiation, all other members of which were assigned to the genus *Engytatus* when the latter group was elevated from subgeneric to generic status by Cassis (1986). The current nomenclatural adjustment thus aligns the species binomial with current taxonomy.

### Engytatus terminalis

Taxon classificationAnimaliaHemipteraMiridae

(Gagné)

Cyrtopeltis terminalis Gagné 1968: 42.
Engytatus terminalis : Schuh 1995: 497.

#### Material examined.

HAWAIIAN ISLANDS, Oahu: 2 males, 2 females, Koolau Mountains, Mt. Tantalus area, gulch crossing on Kaluawahine Trail, 1500 ft [455 m], 21°20'13"N, 157°48'55"W, 23 May 2017, on Cyrtandracf.sandwicensis, CL 8528, D. A. Polhemus (BPBM); 3 males, 3 females, Waianae Mountains, Honouliuli Forest Reserve, South Fork of Kaluaa Gulch, 26 April 1970, on *Cyrtandrachristophersenii* [= *C.waianaeensis* x *C.garnotiana*], W. C. Gagné (BPBM); 1 male, 1 female, Koolau Mountains, Honolulu Watershed Forest Reserve, Kului Gulch, 400 m, 31 January 1971, on *Cyrtandracordifolia* W. C. Gagné (BPBM).

#### Ecologicalnotes.

Three species of Hawaiian *Engytatus*, all of them endemic to Oahu, appear to be exclusively associated with the host-plant genus *Cyrtandra* in the Gesneriaceae, which contains 58 endemic Hawaiian species, all apparently derived from a single colonizing ancestor (Wagner et al. 1999, Cronk et al. 2005). Previously, Gagné (1968) speculated that specialization on individual species within this diverse local plant radiation could be a significant species isolating mechanism within Hawaiian *Engytatus*.

In regard to *E.terminalis*, Gagné (1968) noted its association with *Cyrtandra*, but did not specify which species was involved. Bishop Museum specimens collected subsequent to his study bear host-plant labels indicating that they were taken on *C.cordifolia* Gaudichard. More recently, specimens have been taken on the slopes of Mt. Tantalus, in the Koolau Mountains behind Honolulu, from Cyrtandracf.sandwicensis. The host-plant determination for these latter specimens is provisional because hybrids between *C.sandwicensis* (H. Léveillé) H. St. John & Storey and *C.grandiflora* Gaudichard are known to occur in the Mt. Tantalus area, based on Bishop Museum herbarium specimens, but the large, pubescent leaves of the plants in question are most similar to those of *C.sandwicensis*. It therefore appears that *E.terminalis* occurs on at least two *Cyrtandra* species in the Koolau Mountains. Other Gagné specimens in the Bishop Museum from Kaluaa Gulch, in the Waianae Mountains, are recorded as having been taken on *C.christophersenii* H. St. John & Storey, which is now considered a hybrid of *C.waianaeensis* H. St. John & Storey and *C.garnotiana* Gaudichard (Wagner et al. 1999). *Engytatusterminalis* thus utilizes a minimum of three species of *Cyrtandra* across Oahu as a whole, to some extent invalidating the hypothesis of Gagné (1968) that individual host-plant association would prove to be a species isolating mechanism in the genus.

### Engytatus confusus

Taxon classificationAnimaliaHemipteraMiridae

(Perkins)

Cyrtopeltis confusa Perkins, 1911: 729.
Engytatus confusus : Zimmerman 1948: 189.
Cyrtopeltis (Engytatus) confusa : Carvalho 1958: 185.

#### Material examined.

HAWAIIAN ISLANDS, Oahu: 6 males, 9 females, Waianae Mountains, Mt. Palikea, gulch head NE of summit, 915 m, 21°24'52"N, 158°05'59"W, 28 August 2013, on *Cyrtandrawaianaeensis*, CL 8518, D. A. Polhemus (BPBM); 2 females, Waianae Mountains, Mt. Palikea, head of Palawai Gulch, 845 m, 21°24'46"N, 158°05'59"W, 28 August 2013, on *Cyrtandrawaianaeensis*, CL 8518, D. A. Polhemus (BPBM); 4 males, 4 females, Koolau Mountains, Punaluu Valley, 1000 ft, 28 September 1968, on *Cyrtandrapropinqua*, W. C. Gagné (BPBM); 2 males, Waianae Mountains, Kawaihapoi Gulch, 548 m, 29 September 1971, on *Cyrtandra* sp., W. C. Gagné (BPBM).

#### Ecologicalnotes.

The association of this species with the host-plant *Cyrtandracordifolia* was previously reported by Gagné (1968). Further specimens have been subsequently collected on *C.propinqua* C. N. Forbes and *C.waianaeensis* H. St. John & Storey. *Engytatusconfusus* therefore seems to occur on at least three species of *Cyrtandra* on Oahu, one of which is also utilized by *E.terminalis*. This once again indicates that individual host-plant association is not a strong isolating mechanism for the Oahu *Engytatus* species feeding on *Cyrtandra*.

### Engytatus hawaiiensis

Taxon classificationAnimaliaHemipteraMiridae

(Kirkaldy)

Cyrtopeltis hawaiiensis Kirkaldy, 1092: 138.
Engytatus hawaiiensis Zimmerman, 1948: 1988.
Cyrtopeltis (Engytatus) hawaiiensis : China and Carvalho 1952: 160.

#### Material examined.

HAWAIIAN ISLANDS, Maui: 1 male, 1 female, East Maui, Koolau Forest Reserve, 2042 m, 8 August 1973, on *Dubautia* cf. *coriacea [=D.thyrisiflora*], W. C. Gagné (BPBM); 6 males, 7 females, Haleakala National Park, West Rim, 9600 ft, 12 August 1975, on *Railliardia* [= *Dubautia* sp.], J. W. Beardsley (BPBM).

#### Ecologicalnotes.

This species has been previously recorded as occurring on several species of *Railliardia* (Gagné 1968), a host-plant genus subsequently synonymized within *Dubautia*, in the Asteraceae. Based on these records and examination of other Bishop Museum specimens, *E.hawaiiensis* occurs on *Dubautiamenziesii* (A. Gray) D. D. Keck, *D.platyphylla* (A. Gray) D. D. Keck, and *D.thyrisiflora* (Sherff) D. D. Keck, and thus is not strictly confined to a single host-plant species within this genus.

### Engytatus sidae

Taxon classificationAnimaliaHemipteraMiridae

(Gagné)

Cyrtopeltis sidae Gagné, 1968: 40.
Engytatus sidae : Schuh, 1995: 497.

#### Material examined.

HAWAIIAN ISLANDS, Lanai: 15 males, 8 females, Kaumolu Bay heiau, 7 February 1971, on *Sida* sp., J. W. Beardsley (BPBM).

#### Ecologicalnotes.

This species was originally described from Maui, and the record above demonstrates its occurrence on Lanai as well.

## Supplementary Material

XML Treatment for Engytatus

XML Treatment for Engytatus henryi

XML Treatment for Engytatus kahakai

XML Treatment for Engytatus terminalis

XML Treatment for Engytatus confusus

XML Treatment for Engytatus hawaiiensis

XML Treatment for Engytatus sidae

## Appendix 1

**Table:** Checklist of Hawaiian *Engytatus* species with known host plants and distributions within the Hawaiian Islands.

Species	Host plant(s)	Distribution
*Engytatusconfusus* (Perkins)	*Cyrtandrawaianaeensis* (Gesneriaceae) *Cyrtandrapropinqua* (Gesneriaceae)	Oahu
*Engytatuscyrtandrae* (Gagné)	*Cyrtandra* sp. (Gesneriaceae)	Oahu
*Engytatushawaiiensis* (Kirkaldy)	*Dubautiamenziesii* (Asteraceae) *Dubautiaplatyphylla* (Asteraceae) *Dubautiathyrisiflora* (Asteraceae)	Maui
*Engytatushenryi* sp. n.	*Abutilonsandwicense* (Malvaceae)	Oahu
*Engytatuskahakai* (Asquith)	*Scaevolasericea* (Goodeniaceae)	Kauai, Molokai
*Engytatuslysimachiae* (Carvalho & Usinger)	*Lysimachia* sp. (Primulaceae)	Kauai
*Engytatusperplexa* (Gagné)	*Dubautia* sp. (Asteraceae)	Maui
*Engytatusphyllostegiae* (Carvalho & Usinger)	*Phyllostegia* sp. (Labiatae)	Oahu
*Engytatusterminalis* (Gagné)	Cyrtandracf.sandwicensis (Gesneriaceae) *Cyrtandracordifolia* (Gesneriaceae) *Cyrtandrachristophersenii* (Gesneriaceae)	Oahu
*Engytatussidae* (Gagné)	*Sida* sp. (Malvaceae)	Maui, Lanai
